# Can urine studies be replaced by serum free light chains measurements to assign responses in multiple myeloma patients?

**DOI:** 10.3389/fonc.2022.1056293

**Published:** 2022-11-30

**Authors:** Maria Cruz Cárdenas, Belén Iñigo, Isabel Ortega, Maria Angeles Palomar, Marina Menéndez, Paula Plaza, Mercedes Martínez-Novillo, Celina Benavente

**Affiliations:** ^1^ Department of Clinical Analysis, Institute of Laboratory Medicine, IdSSC Hospital Clínico San Carlos, Madrid, Spain; ^2^ Department of Haematology, Hospital Clínico San Carlos, Madrid, Spain

**Keywords:** serum free light chains, urine protein electrophoresis, urine protein immunofixation, response criteria, multiple myeloma

## Abstract

Serum and urine protein electrophoresis and immunofixation are the preferred techniques for monitoring monoclonal proteins and evaluating treatment response in multiple myeloma (MM) patients with measurable disease. However, urine studies are subjected to limitations that may lead to inaccuracies or prevent guidelines compliance. We retrospectively studied if the substitution of urine studies by measuring serum free light chains (sFLCs) results in a comparable disease monitoring, both in intact immunoglobulin (II) and light chain (LC) MM patients. In our cohort, equal or higher percentages of disease were identified by sFLCs at baseline and maximum response as compared to urine studies. Achieving very good partial response or better (≥VGPR) according to the response criteria proposed by the French group (evaluating sFLCs instead of urine) and the IMWG response criteria were associated to a 62% and 63% reduced risk of progression, respectively. A similar prognostic value for reaching ≥VGPR was also observed among LCMM patients when the French group and the IMWG response criteria were applied. Overall, these results support the replacement of urine studies by the sFLCs assay in IIMM. In LCMM, sFLCs could be used for monitoring and urine studies could be performed only to confirm complete remissions and progressions.

## Introduction

Multiple myeloma (MM) is an incurable monoclonal gammopathy characterized by the proliferation of clonal plasma cells in the bone marrow. These malignant plasma cells secrete intact immunoglobulins and/or free light chains (FLCs) that are immunochemically and electrophoretically homogeneous, which are consequently known as monoclonal proteins (M-proteins). M-proteins can be found in serum (both, intact immunoglobulins and FLCs) and urine (only FLCs), and they are considered as a disease surrogate biomarker. Therefore, the diagnosis, therapy response assessment and monitoring of MM patients rely, partly, on the detection and the measurement of the M-protein (1–4).

In this regard, the International Myeloma Working Group (IMWG) has provided specific directives (2, 3). For most MM patients, serum and urine protein electrophoresis (SPEP/UPEP) and immunofixation electrophoresis (sIFE/uIFE) are the recommended techniques to detect and measure M-proteins at diagnosis and follow-up. Only in patients with unmeasurable disease (serum M-protein <1 g/dL by SPEP and urine M-protein <200 mg/24h by UPEP) at baseline, the IMWG recommends measuring serum FLCs throughout monitoring as long as the involved FLC (iFLC) is above 100 mg/L and the FLC kappa/lambda ratio (FLCr) is altered. Therefore, in those cases in which serum M-protein is below 1 g/dL, but there is measurable disease in urine by UPEP (M-protein >200 mg/24h) and in serum by the FLC assay (iFLC >100 mg/L), follow-up in urine must be prioritized (2, 5).

However, it is well-known that M-protein quantification in urine is associated with several limitations. Firstly, it is influenced by the renal metabolism. Achieving serum concentrations over 133 mg/L of kappa FLCs and over 278 mg/L of lambda FLCs is necessary to overcome the reabsorption capacity of the renal proximal tubule and, consequently, to find M-proteins in urine (6). Moreover, urine samples are often not provided due to the difficulties elderly patients have in collecting them (7) or are not appropriately collected leading to inaccuracies in the quantification of the M-protein (8, 9). Likely because serum FLCs are not affected by these limitations, different studies have shown a poor correlation between 24h-urine studies and sFLCs measurements (10–12). In addition, different reports have demonstrated a clinical benefit in monitoring monoclonal FLCs in serum instead of in urine due to its higher analytical sensitivity and accuracy (8, 9, 13, 14), its superior prognostic value (13), and its higher performance as an early biomarker of progression (15).

Consequently, the French group has recently proposed a modified treatment response criteria in which 24h-urine studies have been replaced by serum FLCs measurement (16).

The aim of this study is to compare the IMWG (2) and the French group response criteria (16) and determinate whether urine test can be replaced by sFLCs without affecting the prognostic value of the different response categories, in both intact immunoglobulin MM (IIMM) and light chain MM (LCMM) patients.

## Methods

### Study design and patients

One hundred newly diagnosed MM patients with paired serum and urine samples at baseline and at maximum response that were treated at the San Carlos Clinical Hospital from January 2008 to December 2020 were included in this retrospective study. Patients with oligosecretory and non-secretory disease were excluded. A total of 110 lines of treatment were evaluated: 80 patients were evaluated during their first line of treatment, 10 during their second line of treatment and 10 during their first and second lines of treatment. All available urine and/or serum samples from the same line of treatment for each patient were evaluated including, at least, one sample before starting treatment (baseline); another sample corresponding to the maximum response achieved [post autologous stem cell (ASCT) in transplanted patients] and a final sample of progression if applicable. The study was approved by the Clinical Research Ethics Committee of the San Carlos Clinical Hospital.

### Laboratory methods

SPEP and UPEP were performed by capillary zone electrophoresis in the Paragon CZE 2000 (Beckman Coulter; Brea, United States) and the Capillarys (Sebia; Evry, France) analytical systems. Diafiltration pre-treatment was applied to urine samples before performing protein electrophoresis to remove interfering substances (17). sIFE and uIFE were performed on the Hydrasys system (Sebia; Evry, France). Serum FLCs were measured by immunonephelometry with the Freelite assay (The Binding Site; Birmingham, UK) on a BN proSpec analyzer (Siemens; Erlangen, Germany). Reference ranges for kappa sFLC were 3.3 to 19.4 mg/L; for lambda sFLC, 5.7 to 26.3 mg/L; and for the serum FLCr, 0.26 to 1.65.

### Response assessment

Response to treatment was assigned according to the IMWG (2) or the French group (16) criteria, both summarized in **Supplementary Table 1**. Given the trend towards starting a new line of treatment before symptoms onset, the relapse from complete response (RCR) category was included. Bone marrow infiltration by malignant plasma cells below 5% was verified in 64% of patients reaching complete remission (CR). In those who did not undergo a bone marrow study, CR was considered when negative sIFE and uIFE results were achieved.

### Statistical analysis

Statistical analyses were performed using the GraphPad Prism v.9.0.0. Progression-Free Survival (PFS) was calculated as time from maximum response until RCR, progression or death from any cause. Survival curves were constructed using the Kaplan-Meier method and the (two-sided) log-rank (mantel-Cox) test for comparison between groups. Concordance between the IMWG and the French group response criteria was performed by quadratic weighted kappa analysis. A kappa value > 0.80 was interpreted as very good agreement.

## Results

### Diagnostic sensitivity and measurable disease

The baseline characteristics for the 100 MM patients included in the study are shown in **Supplementary Table 2**. The median age of the entire cohort was 68 years (range, 36-88). Eighty-two patients had IIMM and 18 had LCMM. Twenty-seven % of patients had International Staging System (ISS) stage I, 33% II and 40% III. Thirty-six % of patients underwent ASCT.

**Figure 1:**
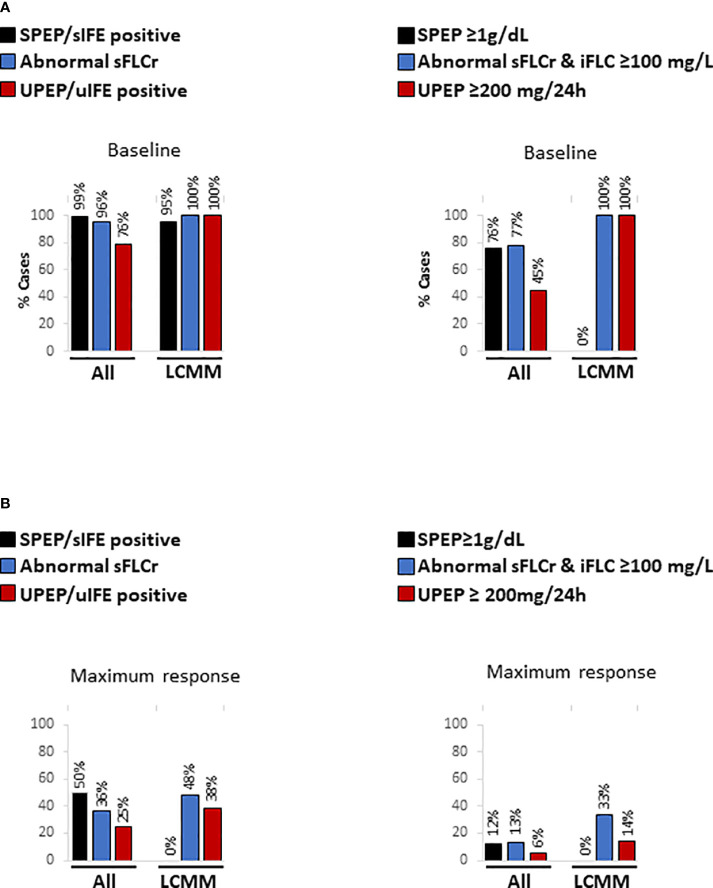
Percentage of detectable and measurable disease by SPEP/sIFE, serum FLCs and UPEP/uIFE tests for both, the entire cohort and LCMM patients **(A)** before starting the first and/or the second line of treatment (baseline) and **(B)** at time of maximum response. LCMM, light chain multiple myeloma; sFLCr, serum free light chains ratio; sIFE, serum immunofixation electrophoresis; SPEP, serum protein electrophoresis; uIFE, urine immunofixation electrophoresis; UPEP, urine protein electrophoresis.

Before starting the first and/or the second line of treatment (baseline), SPEP and/or sIFE were able to detect the presence of a M-protein in 99% of cases (**Figure 1A**). The M-protein was undetectable by SPEP/sIFE in one sample from a LCMM patient. By contrast, an abnormal sFLC ratio was shown by 96% of cases. The 4% baseline samples with normal sFLC ratio corresponded to 5 IIMM patients. Regarding the detection of measurable disease, both serological approaches provided similar results (SPEP>1 g/dL: 76% vs abnormal sFLCr with iFLC>100 mg/L: 77%). UPEP and/or uIFE were the techniques exhibiting lower performance in terms of identifying detectable (positive UPEP/uIFE: 76%) and measurable disease (UPEP>200 mg/24h: 45%) in the entire cohort. When LCMM patients were separately analysed, serum FLCr and UPEP/uIFE detected measurable disease in 100% of cases evaluated. SPEP/sIFE detected a M-protein in almost all patients, but at levels below 1 g/dL.

Subsequently, the same analysis was implemented at time of maximum response, when significantly lower levels of disease were found (**Figure 1B**). As observed at baseline, SPEP/sIFE identified the presence of a M-protein in a higher number of patients than the sFLC ratio in terms of detectable disease (50% vs 36%) but not of measurable disease (12% vs 13%). UPEP and/or uIFE were again the techniques showing lower performance, displaying positive results in 25% of the samples and UPEP over 200 mg/24h in only 6%. Among LCMM patients, at maximum response the sFLC ratio was able to detect higher percentage of both detectable (48% vs 38%) and measurable disease (33% vs 14%) than UPEP/uIFE. At this point, SPEP/sIFE could not detect the presence of disease in any patient.

### Prognostic value of the sFLC ratio and UPEP/uIFE tests

**Figure 2:**
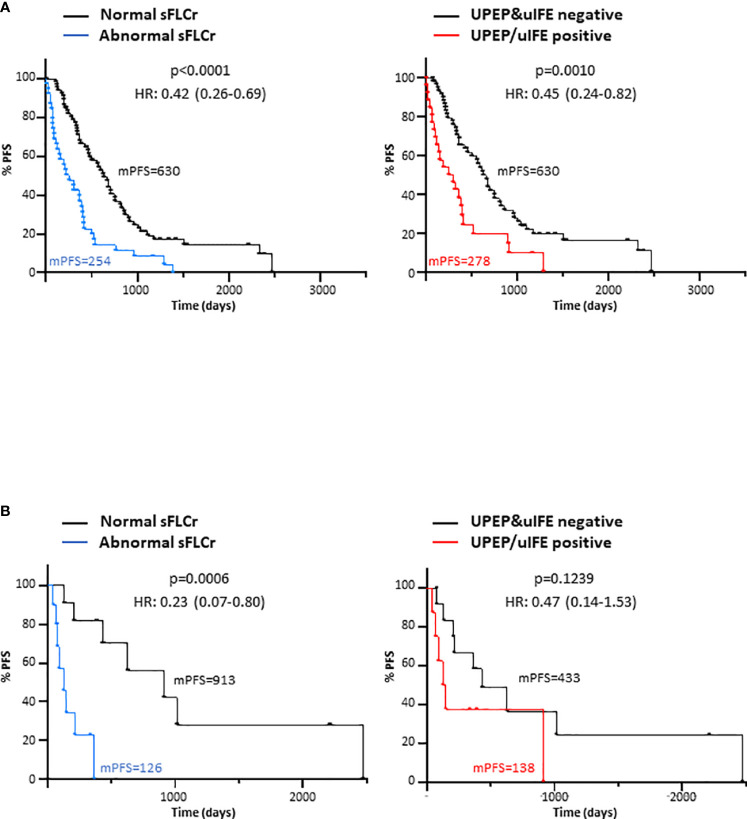
Progression-free survival (PFS) according to the normalized sFLCr and negativized 24-hour urine studies at maximum response for the entire patient cohort **(A)** and for LCMM patients **(B)**. mPFS, median progression-free survival; HR, Hazard ratio; sFLCr, serum free light chains ratio; uIFE, urine immunofixation electrophoresis; UPEP, urine protein electrophoresis.

We next evaluated the prognostic value of normalizing the sFLC ratio and absence of monoclonal protein in the urine at maximum response, irrespective of the results obtained by SPEP/sIFE. For the entire cohort, not detecting the presence of a M-protein in serum by the sFLC ratio [p<0.0001; HR (95%CI)=0.42 (0.26-0.69)] and in urine by UPEP/uIFE [p=0.0010; HR (95%CI)=0.45 (0.24-0.82)] translated in a significant prolonged PFS (**Figure 2A**). The median PFS (mPFS) for patients with normal sFLC ratio doubled that from patients with abnormal sFLC ratio (630 vs 254 days). Likewise, a two-fold increase in the mPFS of patients negative for UPEP/uIFE as compared with that of patients with detectable M-protein in urine (630 vs 278 days) was observed.

In concordance with the entire cohort, a normal sFLC ratio was also associated with a benefit in terms of PFS in LCMM patients [p=0.0006; HR (95%CI)=0.23 (0.07-0.80)] as observed in **Figure 2B**. Notably, patients with normal sFLC ratio showed an mPFS seven times higher than patients with abnormal sFLC ratio (913 vs 123 days). However, patients with a detectable M-protein in urine did not exhibit a shorter PFS as compared to those UPEP/uIFE negative (p=0.1239).

### Response assessment by the IMWG or French group criteria

**Figure 3:**
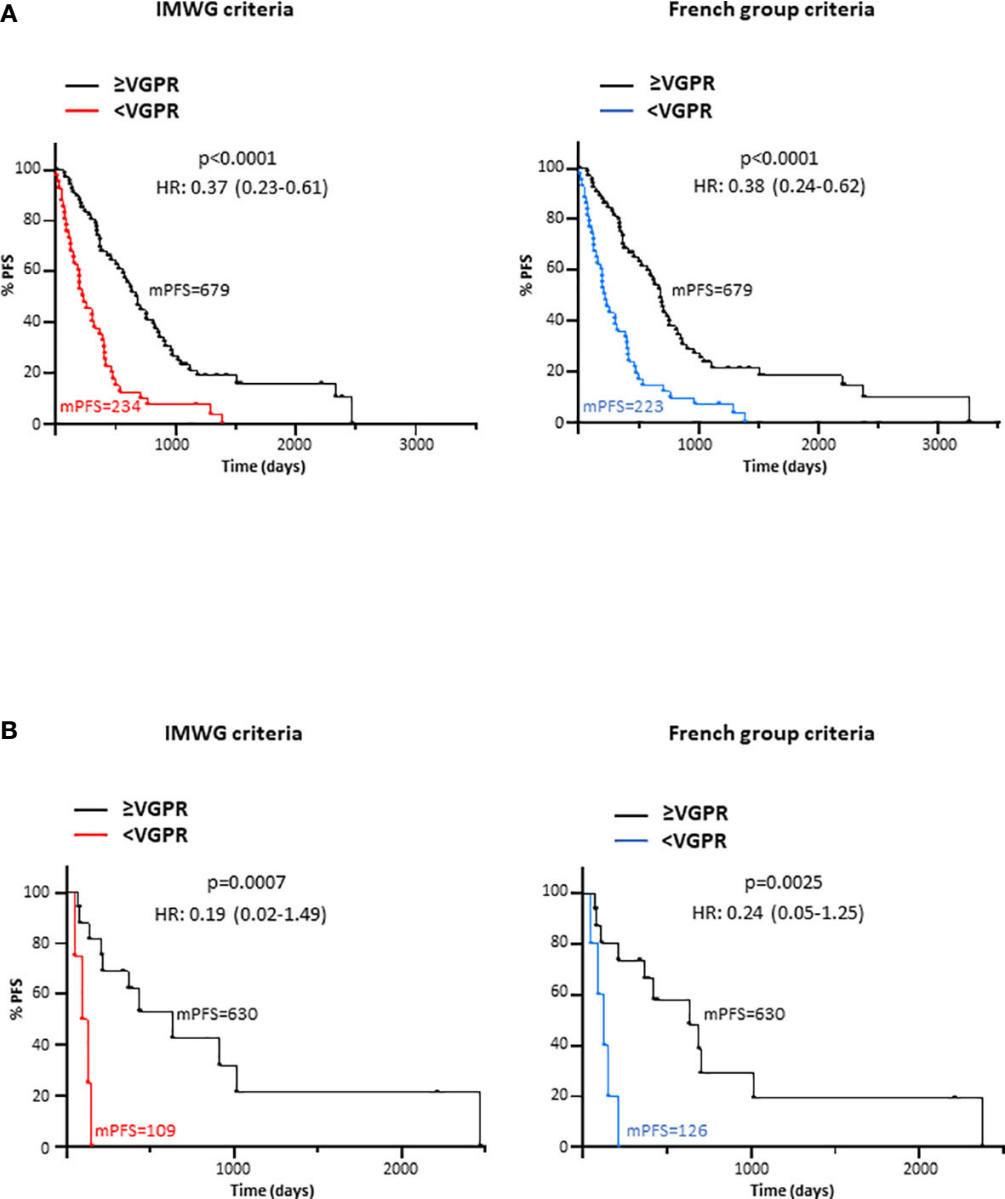
Progression-free survival (PFS) according to the depth of response (≥VGPR or <VGPR) achieved at maximum response attending to the IMWG and the French group criteria in **(A)** the entire cohort and **(B)** only in LCMM patients. HR, Hazard ratio; IMWG, International Myeloma Working Group; mPFS, median progression-free survival; VGPR, very good partial response.

The concordance between the conventional IMWG response criteria based on SPEP/sIFE and UPEP/uIFE, and the modified response criteria proposed by the French group based on SPEP/sIFE and sFLCs was assessed. In all patients (**Table 1**) an 86% of agreement was observed between both approaches when assessed at maximum response, leading to a very high correlation as indicated by the 0.9 (95%CI=0.68-1.00) kappa with quadratic weighting obtained. Similar results were observed when all the available samples from baseline to progression, RCR or last follow-up for all the evaluated lines of treatment (n=294) were analysed (**Supplementary Table 3**). Then, patients were grouped attending to the depth of response achieved at maximum response in ≥VGPR or <VGPR by both, the IMWG and the French group criteria, for survival analysis. As seen in the **Figure 3A**, almost identical results were obtained. Patients reaching ≥VGPR by both response criteria exhibited a significant longer PFS than those in<VGPR [IMGW: p<0.0001; HR (95%CI)=0.37 (0.23-0.61) and French group: p<0.0001; HR (95%CI)=0.38 (0.24-0.62)].

**Table 1:** Concordance between maximum responses assigned by the IMWG criteria and the French group criteria in all patients and only in LCMM patients.

All patients									
		IMWG criteria							
		RCR	PD	SD	MR	PR	VGPR	CR	Total
**French group criteria**	RCR	**0**	0	0	0	0	0	0	0
	PD	0	**1**	0	0	0	0	0	1
	SD	0	0	**1**	2	1	0	0	4
	MS	0	0	0	**4**	1	0	0	5
	PR	0	1	0	2	**28**	1	1	33
	VGPR	0	0	0	0	0	**17**	4	21
	CR	0	0	0	0	0	3	**43**	46
Total		0	2	1	8	30	21	48	110
**Concordance (%)**		**100**	**50**	**100**	**50**	**93**	**81**	**90**	**86**
***Kappa with Quadratic Weighting (IC 95%)= 0.9 (0.68-1.00)***									
**LCMM patients**									
		**IMWG criteria**							
		**RCR**	**PD**	**SD**	**MR**	**PR**	**VGPR**	**CR**	**Total**
**French group criteria**	RCR	**0**	0	0	0	0	0	0	0
	PD	0	**0**	0	0	0	0	0	0
	SD	0	0	**0**	0	1	0	0	1
	MR	0	0	0	**1**	0	0	0	1
	PR	0	0	0	2	**0**	0	1	3
	VGPR	0	0	0	0	0	**2**	3	5
	CR	0	0	0	0	0	2	**9**	11
Total		0	0	0	3	1	4	13	21
**Concordance (%)**		**100**	**100**	**100**	**33**	**0**	**50**	**69**	**57**
***Kappa with Quadratic Weighting (IC 95%)= 0.7 (0.21-1.00)***									

CR, complete response; IMWG, International Myeloma Working Group; MR, minimal response; PD, progressive disease; PR, partial response; RCR, relapse from complete response; SD, stable disease; VGPR, very good partial response. The bold values are the number of coincidences.

Finally, only LCMM patients were evaluated. As compared to the entire cohort, the concordance between the IMWG and the French group response criteria was lower [kappa with quadratic weighting (95%CI)= 0.7 (0.21-1.00)]. Only a 57% of agreement was observed for LCMM patients (**Table 1**). The concordance between both response criteria did not substantially increase when all the available samples from baseline to progression, RCR or last follow-up for the evaluated lines of treatment (n=57) were analysed (**Supplementary Table 3**). Despite the existence of discordances, patients classified as in ≥VGPR shown a significantly longer PFS as compared to those in <VGPR when using both the IMWG [p=0.0007; HR (95%CI)=0.19 (0.02-1.50)] and the French group [p=0.0025; HR (95%CI)=0.24 (0.05-1.25)] response criteria (**Figure 3B**).

## Discussion

In the last few years several studies have been questioning the need for monitoring the M-protein in urine, especially in patients who secrete monoclonal intact immunoglobulins. These studies have been motivated by the aforementioned limitations associated to 24-hour urine studies that often prevent guidelines compliance.

In this regard, some reports have demonstrated that there are no differences in terms of survival between IIMM patients in CR with negative uIFE and those in which uIFE results were not available (18, 19). Other publications go further and propose to replace 24h-urine studies by sFLCs measurements, based on the superior analytical sensitivity and the higher prognostic value demonstrated by serum FLCs. Dejoie and colleagues observed higher percentages of both LCMM and IIMM patients with elevated iFLC as compared to those with positive UPEP analysis at baseline, during induction and after ASCT. In addition, an increased proportion of patients with measurable serum FLCs disease with respect to those with measurable disease in urine was observed at the same time points (13, 14). In our cohort, generally, a higher percentage of IIMM and LCMM patients presented abnormal serum FLCr over UPEP/uIFE positive results, both at baseline and at maximum response. Similar results were observed when measurable serum FLCs and urine disease were evaluated. Regarding the prognostic value, Sencar et al. evaluated 78 MM patients who relapsed after transplant. No relapse was missed when serum studies (SPEP, sIFE and sFLCs) were performed and uIFE analysis was omitted (20). Dejoie et al. evaluated the ability of the iFLC, serum FLCr, UPEP and uIFE to distinguish two populations with different PFS among 113 LCMM patients at the end of induction therapy. Contrary to patients with positive UPEP or uIFE, those with elevated iFLC or abnormal sFLCs were associated to a significant shorter PFS (13). Here, although in a lower number of LCMM patients, we report similar results. LCMM patients with abnormal serum FLCr at the time of maximum response exhibited a significant shorter PFS as compared to those with normal serum FLCr. However, survival analysis did not reach statistical significance for LCMM patients with positive versus negative UPEP or uIFE results. When the entire cohort, including IIMM and LCMM patients, was evaluated both serum FLCr and UPEP/uIFE status demonstrated to have prognostic value in terms of PFS.

Considering all the previous findings we aimed to compare the modified response criteria proposed by the French group (16) with the standard IMWG response criteria. The agreement between both methods was about 85% when assessing only the maximum response and all responses available from baseline to progression, RCR or the last follow-up. This result was in concordance with the 81% of agreement found by Dejoie et al. (14). In addition, we also evaluated the concordance between both criteria only in LCMM patients. As expected, since SPEP and sIFE are not considered in these patients, the percentage of agreement in this population was lower (about 60%). Subsequently, patients were grouped in ≥VGRP or <VGPR for survival analysis. In the entire cohort and only in patients with LCMM, responses ≥VGRP were associated to a significant longer PFS as compared to inferior outcomes, irrespective of the response criteria employed. However, when LCMM patients are grouped in ≥CR or <CR (**Figure 2B**), only the modified criteria proposed by the French group distinguished two populations with significant different PFS.

In conclusion, our results indicate that the French group modified response criteria in which 24h-urine test were replaced by serum FLCs exhibits a similar prognostic value compared with standard IMWG response criteria in both, IIMM and LCMM patients. Given the greater feasibility of serum FLCs measurement, this assay could replace 24h-urine test in all IIMM patients. In LCMM, serum FLCs could be employed for monitoring and 24h-urine studies could be performed only to confirm CRs and progressions.

## Data availability statement

The raw data supporting the conclusions of this article will be made available by the authors, without undue reservation.

## Ethics statement

The studies involving human participants were reviewed and approved by Clinical Research Ethics Committee of the San Carlos Clinical Hospital. Written informed consent for participation was not required for this study in accordance with the national legislation and the institutional requirements.

## Author contributions

MC administrated the project, designed the research, collected data, performed statistical analysis, and wrote the manuscript. BI designed the research, collected data, performed statistical analysis, and reviewed the manuscript. IO contributed to data collection and reviewed the manuscript. MP contributed to data collection and reviewed the manuscript. MM contributed to data collection and reviewed the manuscript. PP contributed to data collection and reviewed the manuscript. MM-N critically reviewed the manuscript. CB critically reviewed the manuscript. All authors contributed to the article and approved the submitted version.

## Funding

This work was supported by a grant from the José Luis Castaño-SEQC^ML^ Foundation that belongs to the Spanish Society of Laboratory Medicine SEQC-ML, Spain.

## Conflict of interest

The authors declare that the research was conducted in the absence of any commercial or financial relationships that could be construed as a potential conflict of interest.

## Publisher’s note

All claims expressed in this article are solely those of the authors and do not necessarily represent those of their affiliated organizations, or those of the publisher, the editors and the reviewers. Any product that may be evaluated in this article, or claim that may be made by its manufacturer, is not guaranteed or endorsed by the publisher.
