# Supplementary material for: Can urine studies be replaced by serum free light chains measurements to assign responses in multiple myeloma patients?
Source: Front Oncol. 2022 Nov 30;12:1056293. doi: 10.3389/fonc.2022.1056293 (PMC9748693; doi:10.3389/fonc.2022.1056293)
Supplement: Supplementary file 1 [file DataSheet_1.pdf]

Supplementary Table 1. Response criteria evaluated in this study

Response category	IMWG criteria	French group criteria
Stringent complete response (sCR)	CR as defined below plus normal sFLCr and absence of clonal cells in BM by immunohistochemistry or 2–4-colour flow cytometry	CR as defined below plus absence of clonal cells in BM by immunohistochemistry or 2–4-colour flow cytometry
Complete response (CR)	Negative sIFE and uIFE, disappearance of any soft tissue plasmacytomas and ≤5% plasma cells in BM	Negative sIFE, normal sFLCr, disappearance of any soft tissue plasmacytomas and ≤5% plasma cells in BM
Very good partial response (VGPR)	Serum and urine M-protein detectable by IFE but not on SPEP/UPEP or ≥90% reduction in serum M-protein plus urine M-protein level < 100 mg/24 h	Serum M-protein detectable by sIFE but not on SPEP or ≥90% reduction in serum M-protein and abnormal sFLCr and ≥90% reduction in dFLC levels
Partial response (PR)	≥50% reduction of serum M-protein and reduction in 24-h urinary M-protein by ≥90% or to <200 mg/24 h	≥50% reduction of serum M-protein and abnormal FLC ratio and ≥50% reduction in dFLC levels
Stable disease (SD)	Not meeting criteria for CR, VGPR, PR, PD or RCR	Not meeting criteria for CR, VGPR, PR, PD or RCR
Progressive disease (PD)	Requires any one or more of the following:<br>• Increase of ≥25% from lowest response in serum M-protein (the absolute increase must be ≥5 g/L) and/or urine M-protein (the absolute increase must be ≥200 mg/24 h).<br>• Only in patients without measurable serum and urine M-protein levels: dFLC levels (the absolute increase must be >100 mg/L)<br>• Only in patients without measurable serum and urine M-protein levels and without measurable disease by FLC levels: BM plasma cell percentage (absolute percentage must be ≥10%)<br>• Definitive development of new bone lesions or soft tissue plasmacytomas or definite increase in the size of existing bone lesions or soft tissue plasmacytomas<br>• Development of hypercalcaemia (corrected serum calcium >11.5 mg/dL or 2.65 mmol/L) that can be attributed solely to the plasma cell proliferative disorder	Requires any one or more of the following:<br>• Increase of ≥25% from lowest response in serum M-protein (the absolute increase must be ≥5 g/L) and/or dFLC levels (the absolute increase must be >100 mg/L)<br>• BM plasma cell percentage (the absolute % must be ≥10%)<br>• Definitive development of new bone lesions or soft tissue plasmacytomas or definite increase in the size of existing bone lesions or soft tissue plasmacytomas<br>• Development of hypercalcaemia (corrected serum calcium >11.5 mg/dL or 2.65 mmol/L) that can be attributed solely to the plasma cell proliferative disorder
Relapse from complete response (RCR)	Any one or more of the following criteria:<br>• Reappearance of serum or urine M-protein by immunofixation or electrophoresis<br>• Development of ≥5% plasma cells in the BM<br>• Appearance of any other sign of progression (ie, new plasmacytoma, lytic bone lesion, or hypercalcaemia see above)	Any one or more of the following criteria:<br>• Reappearance of M-protein by SPEP, sIFE or abnormal sFLCr<br>• Development of ≥5% plasma cells in the BM<br>• Appearance of any other sign of progression (ie, new plasmacytoma, lytic bone lesion, or hypercalcaemia see above)

BM: bone marrow; dFLC: the difference between involved and uninvolved serum free light chains; M-protein: monoclonal protein; sFLCr: serum free light chain ratio; sIFE: serum immunofixation electrophoresis; SPEP: serum protein electrophoresis; uIFE: urine immunofixation electrophoresis; UPEP: urine protein electrophoresis.

Supplementary Table 2. Patient characteristics at diagnosis.

		N=100
**Median age, years (range)**		68 (36-88)
**Sex, n (%)**		
	Male	54 (54)
	Female	46 (46)
**ISS, n (%)**		
	I	27 (27)
	II	33 (33)
	III	40 (40)
**Immunotype, n (%)**		
	IgG	55 (55)
	IgA	25 (25)
	IgM	1 (1)
	IgD	1 (1)
	kappa	7 (7)
	lambda	11 (11)
**Treatment, n (%)**		
	ASCT	36 (36)
	No ASCT	64 (64)

ASTC: Autologous stem cell transplantation; ISS: International Stage System.

Supplementary Table 3. Concordance between all the evaluated responses assigned by the IMWG criteria and the French group criteria in all patients and only in LCMM patients.

All patients

		IMWG criteria							
		RCR	PD	SD	MR	PR	VGPR	CR	Total
**French group criteria**	RCR	**21**	0	0	0	1	1	3	26
	PD	6	**58**	0	1	0	0	0	65
	SD	0	0	**4**	2	1	1	0	9
	MR	0	0	0	**8**	1	0	0	9
	PR	0	1	1	2	**56**	3	1	65
	VGPR	0	0	0	1	1	**35**	6	42
	CR	4	0	0	0	0	6	**69**	79
Total		31	59	5	14	60	46	79	294
**Concordance (%)**		**68**	**98**	**80**	**57**	**93**	**76**	**87**	**85**
Kappa with Quadratic Weighting (IC 95%)= 0.9 (0.78-0.96)									

LCMM patients

		IMWG criteria							
		RCR	PD	SD	MR	PR	VGPR	CR	Total
**French group criteria**	RCR	**3**	0	0	0	0	1	2	6
	PD	2	**10**	0	0	0	0	0	12
	SD	0	0	**0**	0	1	0	0	1
	MR	0	0	0	**1**	0	0	0	1
	PR	0	0	1	2	**1**	1	1	7
	VGPR	0	0	0	0	1	**5**	5	10
	CR	2	0	0	0	0	2	**16**	20
Total		7	10	1	3	3	9	24	57
**Concordance (%)**		**43**	**100**	**0**	**33**	**33**	**56**	**67**	**63**
Kappa with Quadratic Weighting (IC 95%)= 0.7 (0.45-0.91)									

CR: complete response; IMWG: International Myeloma Working Group; MR: minimal response; PD: progressive disease; PR: partial response; RCR: relapse from complete response; SD: stable disease; VGPR: very good partial response.
